# Increased Odds for Depression and Antidepressant Use in the Inactive Spanish Population

**DOI:** 10.3390/ijerph19052829

**Published:** 2022-02-28

**Authors:** Carmen Galán-Arroyo, Damián Pereira-Payo, Jorge Rojo-Ramos, Miguel A. Hernández-Mocholí, Eugenio Merellano-Navarro, Jorge Pérez-Gómez, Ángel Denche-Zamorano, Jose Carmelo Adsuar

**Affiliations:** 1 Promoting a Healthy Society Research Group (PHeSO), Faculty of Sport Sciences, University of Extremadura, 10003 Caceres, Spain; magaar04@alumnos.unex.es (C.G.-A.); jadssal@unex.es (J.C.A.); 2 Health Economy Motricity and Education (HEME), Faculty of Sport Science, University of Extremadura, 10003 Caceres, Spain; dpereirab@alumnos.unex.es (D.P.-P.); jorgepg100@unex.es (J.P.-G.); 3 Social Impact and Innovation in Health (InHEALTH), University of Extremadura, 10003 Caceres, Spain; jorgerr@unex.es; 4 Physical Activity and Quality of Life Research Group (AFYCAV), Faculty of Sport Science, University of Extremadura, 10003 Caceres, Spain; mhmocholi@unex.es; 5 Facultad de Educación, Universidad Autónoma de Chile, Talca 3460000, Chile; emerellanon@uautonoma.cl

**Keywords:** health, depression, physical activity, antidepressants, sedentary lifestyle, active population, inactive population

## Abstract

Introduction: Depression is a disabling mental illness and therefore also a serious public health problem. It affects 5% of the adult population in the world and is the leading cause of disability, with an annual cost of USD one trillion. In Spain, its prevalence is 13.4%, costing EUR 6000 million a year. Physical inactivity has been linked to an increase in depressive symptoms, with physical activity associated with an improvement in health-related quality of life. Objective: To calculate the odds ratio (OR) and relative risk (RR) of suffering from depression and taking antidepressants in the inactive Spanish population compared to groups with a higher level of physical activity. Method: 17,141 individuals aged 18–69 years residing in Spain and interviewed in the 2017 Spanish National Health Survey were included in this cross-sectional investigation. Results: Dependence relationships were found between the level of physical activity and the prevalence of depression and taking antidepressants (*p* < 0.001). We found elevated ORs and RRs for depression and antidepressant use in inactive people compared to those with a high/very high level of physical activity (Depression: OR: 4.32. CI95%: 3.35–5.57. RR: 1.59. 95% CI: 1.51–1.68; Antidepressants: OR: 4.95. CI95%: 3.59–6.82. RR: 1.61. CI95%: 1.52–1.71). Conclusions: Belonging to an inactive population group increases the risk of suffering from depression and of taking antidepressants.

## 1. Introduction

According to the WHO, 322 million people in the world suffer from depression [1]. This mental illness presents with a disability that affects different areas of life and is associated with increased morbidity and mortality [2,3]. Depression is characterized by significant sadness, listlessness, feelings of guilt or lack of self-esteem, sleep or appetite disorders, feelings of tiredness and lack of concentration over prolonged periods of time [4], and it is the leading cause of disability worldwide, costing USD one trillion in productivity annually [5]. In Spain, its prevalence is 13.4% of the population over 15 years of age (9.1% women; 4.3% men), reaching a cost of EUR 6000 million per year, according to data from the Ministry of Health in 2018. In addition, productivity losses due to work and premature sick leave represent 67% of the total cost of depression in Spain (White Paper on Depression and Suicide 2018). Therefore, it is at minimum a significant public health problem, if not also a social and economic problem [5].

Different strategies are being carried out to decrease the costs of depression, because antidepressants, in addition to being expensive, are increasingly ineffective [6] and physical activity (PA) is being presented as a simple and effective alternative proposal [7]. In this sense, physical inactivity is associated with an increased risk of chronic disease and premature death [8], and sedentary behavior (SB) may be a risk factor for depression [9]. It appears that low physical performance is a predictor of future onset of depression in older adults [10,11]. Almost one third of the world′s population is inactive [12]. Five percent of the world′s population dies due to physical inactivity [13], which is a risk factor for diseases such as cancers, obesity, diabetes, hypertension, Alzheimer′s disease, depression, etc. [14]. Four out of ten Spaniards declare themselves sedentary [13]. This is a cause for concern.

According to the latest meta-analyses, a correlation between PA and improved health and quality of life can be observed [15]. Moreover, PA has a strong influence on the well-being of the individual [16]. It may decrease depression and, as a result, medication intake [17,18]. PA has also been found to protect against the onset of depression regardless of age and geography [19,20]. It may be a prevention and/or treatment strategy for depression [21,22].

At this point, the aim of our study is to calculate the odds ratio (OR) and relative risk of suffering from depression and taking antidepressants in the Spanish inactive population versus groups with a higher level of physical activity. The hypothesis was that physical inactivity increases the risk of suffering from depression and taking antidepressants in the Spanish population.

## 2. Materials and Methods

### 2.1. Study Design

Based on data obtained from public files provided by the Spanish Ministry of Health, Consumer Affairs and Social Welfare (MSCBS), referring to the 2017 Spanish National Health Survey (ENSE 2017), adult questionnaire (Ministry of Health, 2017), this research consisted of a cross-sectional survey-based study.

The ENSE is a survey elaborated every 5 years by the MSCBS in collaboration with the Spanish National Institute of Statistics (INE), which collects information on the health status of the population residing in Spain over 15 years of age. The surveys were conducted during the period between October 2016 and October 2017 and were carried out by qualified, previously trained interviewers.

### 2.2. Participants

The ENSE 2017 had a participation of 23,089 people over 15 years of age who are residents in Spain. This sample was configured based on a random three-phase sampling system.

Of the 23,089 people who participated in the ENSE 2017, only 17,141 were taken into account. The inclusion criteria were persons of legal age in Spain who presented all the data of the study variables, namely, PA variables (Q.113–Q.117), self-perceived health (G.21), variables referring to depression (G.25.1 and G.25.c) and variables referring to taking medication and antidepressants (Q.85, q86, Q.87.a and Q.87.c). Therefore, subjects under 18 years of age were excluded, as they are considered minors in Spain. Those over 70 years of age were also excluded, as the 2017 NSS did not ask such persons about PA performed. Persons who answered “Don′t know/No answer” (NS/NC) to the items mentioned above in the inclusion criteria were also excluded.

### 2.3. Ethics

Due to the characteristics of the research and given that the data were obtained from non-confidential open-access public files published by the MSCBS, the supervision and authorization of any official ethics committee was not necessary.

Regulation (EU) 2016/679 of the European Parliament and Council of 27 April 2016 on the protection of natural persons does not consider this type of public file as likely to be confidential. The data is presented in encrypted form, and therefore respects the anonymity of the participants.

### 2.4. Variables and Procedures

This research required the following questions: sex, age, G21 (Self-perceived health: In the last twelve months, would you say that your state of health has been: very good, good, fair, bad, very bad?), G25_20a (Depression: Do you suffer or have you ever suffered from depression?), G25_20c (Diagnosis of depression: Has a doctor told you that you suffer from it?), Q85 (Prescribed medications: During the past 2 weeks, have you taken any medications that were prescribed by a doctor?), Q86 (Non-prescribed medications: During the past 2 weeks, have you taken any medications, including herbal medications or vitamins, that were not prescribed by a doctor?), Q87_14a (Antidepressants: Next, I am going to read you a list of types of medications, please tell me which one(s) you have taken in the last 2 weeks: antidepressants, stimulants?), Q87_14c (Prescribed antidepressants: Prescribed by a doctor?), Q113 (Intense PA: how many days did you do intense PA?), Q114 (Duration Intense PA: How much time did you spend in total on intense PA?), Q115 (Moderate PA: How many days did you perform moderate PA?), Q116 (Duration Moderate PA: How much time did you spend in total on moderate PA?), Q117 (Walking: Now think about the time you spent walking in the last 7 days) from the ENSE 2017.

With the data obtained in the previous items of the ENSE 2017, the participants were grouped by:

Sex (Men; Women).

Age (Youth (18–34 years); Young adults (35–49 years); Older adults (50–64 years); Older (65–69 years) [23]).

Depression (Depression (Responded “Yes” to questions: Q.25_20a and Q.25_20c). No Depression (If they answered “Yes” to questions: Q.25_20a (“No”), or Q.25_20a (“Yes”) with Q.25_20c (“No”)).

Antidepressants (Antidepressants (If Q.87_14a (“Yes”). No Antidepressants (If Q.87_14a (“No”), or with Q.87_14a (No data) with Q.85 and Q.86 (“No”)).

Self-perceived health (Negative (Responded to G.21: Bad or very bad). Fair (Responded to G21: “Fair”). Positive (G21: “Good” or “Very good”)).

Level of physical activity (NAF) (To the answers given to the questions: Q.113, Q.114, Q.115 and Q.116 of the International Physical Activity Questionnaire (Craig et al., 2003), some factors were applied, adapting the Physical Activity Index [24]).

Intense intensity factor (Fii). A factor of 10 was applied to question P.113, because it is intense PA [24].

Intense frequency factor (Ffi). Factors 0 (No day a week), 1 (One day a week), 2 (Two or three days a week) and 3 (More than three days a week) were applied to the answers given to Q.113 [24].

Factor intense duration (Fdi): Factors 1 (Less than 30 min) and 1.5 (30 or more minutes) were applied to the answers given to Q.114 [24].

Factor moderate intensity (Fim): Factor 5 was applied to question P.115, for moderate PA [24].

Moderate frequency factor (Ffm): Factors 0 (No day per week), 1 (One day per week), 2 (Two or three days per week) and 3 (More than three days per week) were applied to the answers given to Q.115 [24].

Moderate duration factor (Fdm): Factors 1 (Less than 30 min) and 1.5 (30 or more minutes) were applied to the answers given to Q.116 [24].

IAF = (Fii × Ffi × Fdi) + (Fim × Ffm × Fdm).

The IAF could take values from 0 to 67.5 depending on the PA performed. Subsequently, the IAF percentiles obtained from the population were calculated and grouped by NAF: High/Very High (IAF > 30, corresponding to people in a population percentile above the 90th percentile of the IAF); Low/Medium (IAF between 1 and 30, corresponding to a value less than or equal to the 90th percentile of the population′s IAF); Walkers (FWI = 0; responded to question Q.117 that they walked at least one day a week for more than 10 min or more in a row); and Inactive (Individuals with FWI = 0; responded “No day more than 10 min in a row” to Q.117.

### 2.5. Statistical Analysis

The normality of the data of the variables of interest was studied with a Kolgomorov–Smirnov test without finding sufficient evidence to assume normality. Therefore, in the descriptive analysis, to characterize the sample, the data were presented through the median and interquartile range (Continuous variable: Age) and absolute and relative frequencies (Categorical variables: Age group, Depression, Antidepressants, Self-perceived health and PA level). Non-parametric statistical tests were performed, analyzing possible differences between the sexes through the Mann–Whitney U test (Age), and dependency relationships between the categorical variables of interest were analyzed through the chi-square statistic, checking for possible differences between proportions with a pairwise z test for independent proportions and using the Bonferroni correction when necessary. Odds ratios (OR) and relative risks (RR) of perceiving negative health, suffering from depression and taking antidepressants were calculated according to the different groupings of ordinal variables. A correlation study was carried out calculating Spearman′s rho. For all this, a significance level of less than 0.05 was established. The IBM SPSS Statistics v.25 computer program was used for all these analyses.

## 3. Results

No significant differences were found between men and women with regard to the medians presented (*p* = 0.506), which were 47 years both in the general population and in both sexes. Neither were dependency relationships found between sex and age groups (*p* = 0.242): the age groups between 35–49 and 50–64 years were those with the highest representation in both sexes (Table 1).

A total of 9.2% of the population reported having been diagnosed with depression, and significant differences were found (*p* < 0.05) between the proportions of men (5.9) and women (12.2). Another finding was the existence of dependency relationships between the prevalence of depression and sex (*p* < 0.001). Similarly, significant differences were found (*p* < 0.05) between the proportions of antidepressant use in men (3.3) and women (7.6), with an overall prevalence of 5.6% of antidepressant use in the population. A relationship of dependence between sex and antidepressant use was also found (*p* < 0.001). On the other hand, dependency relationships were found between sex on the one hand and self-perceived health (*p* < 0.001) and PA levels (*p* < 0.001) on the other (Table 1).

The self-perceived health status of the general Spanish population presented dependency relationships with the condition of having been, or not having been, diagnosed with depression (*p* < 0.001). Differences were found in the prevalence of the different self-perceived health states in persons with and without a diagnosis of depression (*p* < 0.05). Seventy-eight percent of the population not diagnosed with depression assumed positive health, compared to 28.1% in those diagnosed with depression. In contrast, the states of fair and negative presented a higher prevalence in people with depression (42.8% and 29.1%) than in those not diagnosed (17.6% and 4.5%). The same dependency relationships were found in all PA level groups (*p* < 0.001). Likewise, significant differences were found between the prevalence of the different self-perceived health states between depressives and non-depressives (*p* < 0.05). In depressives, a higher prevalence of positive health was found in those with the highest levels of PA (around 37% in the Low/Medium and High/Very High categories), and the lowest prevalence was found in inactive persons (17.5%), with differences of up to 20 percentage points and reaching up to 30 percentage points in the prevalence of negative health between the inactive (45.6%) and high/very high (13%) levels. The same trend was found in non-depressives (Table 2).

Dependency relationships were found between the prevalence of depression and age group in the general Spanish population, as well as in men and women (*p* < 0.001). (Table 3). In men, significant differences (*p* < 0.05) were found between the proportions of depressives among young people (2.7%), young adults (4.8%) and older adults (7.7% and 9.2%, respectively), with 6.5 proportional points between young men and older men. In women, differences (*p* < 0.05) were found between the proportions of all age groups, with a difference of 17.6 percentage points between young women and older women. These differences in intergroup proportions were also found in the general population (*p* < 0.05). Similarly, there were also dependency relationships between age group and the prevalence of taking antidepressants, both in the general population and in men and women (*p* < 0.001). In men, differences in proportions (*p* < 0.05) were found between youth (1.4%) and young adults (2.9%), and between both groups and older adults (4.5%) and seniors (4.3%), a difference of up to 3 percentage points. In women, these differences were found between the proportions of all age groups (*p* < 0.05), going from 2.2% in young women to 14.7% in older women, a difference of more than 12 percentage points. The same was found in the general population, with statistically significant differences in proportions between all age groups (*p* < 0.05) (Table 3).

The prevalence of depression also showed dependence relationships with PA level, both in the general population and in men and women (*p* < 0.001). In men, the prevalence of depression was 10.2% in inactive persons, presenting differences in proportions with the rest of the PA level groups (*p* < 0.05), with lower proportions with increasing PA level. These proportions were lowest in the high/very high PA group (2.7%), with a difference of 7.5 percentage points between both groups. In women, the prevalence of depression was lower in the higher PA level groups, being 5.6% in women with high/very high PA levels, 8.4% in the low/medium levels and 13.5% and 17.9% in walkers and inactive persons, respectively (*p* < 0.05), a difference of up to 12.3 percentage points. These intergroup differences were also found in the general population (*p* < 0.05). Furthermore, dependency relationships were found between the level of PA and the prevalence of antidepressant use in the general population and in both sexes (*p* < 0.001). Differences in depression prevalence proportions were found between inactive persons (6.7%) and walkers (3.8%), and between these groups and those with higher levels of PA (2.0% and 1.7% in the low/medium and high/very high levels) among men (*p* < 0.05). The same intergroup differences were found in women (*p* < 0.05), with a prevalence of 13.1% in inactive persons, 8.2% in walkers and a prevalence of 4.7% and 3.2% in those with low/medium and high/very high levels of PA, respectively. In the general population, differences were found between the proportions of the four PA level groups (*p* < 0.05) (Table 4).

In the general population, as well as in all age groups, dependency relationships were also found between the prevalence of depression and PA level (*p* < 0.001). In young people, no differences in proportions were found between the inactive (5.5%), walking (4.6%) and low/medium PA level (3.3%) groups, although they were found between these levels and the high/very high level (1.1%) (*p* < 0.05). In the rest of the age groups, differences were found between inactive persons and walkers, and between these and those at higher levels (*p* < 0.05). In the elderly, the differences in proportions reached 16.3 percentage points, the greatest difference in all age groups. When analyzing the relationships between the level of PA and the prevalence of taking antidepressants, dependence relationships were also found in the general population and in all age groups (*p* < 0.001). In young people, differences were found between the proportions of inactive persons and those with the other remaining PA levels (*p* < 0.05). In young adults, differences in proportions were found between all age groups, going from a prevalence of 7.8% in inactive persons to 1.2% in those with a high/very high PA level (*p* < 0.05) (Table 5).

Increased OR and RR of perceiving a negative state of health were found in people with depression versus non-depressives (OR: 8.80. CI: 7.10–10.05; RR: 6.53. CI: 5.87–7.26) and in people who took antidepressants versus those who did not (OR: 9.92. CI: 8.53–11.54. RR: 6.84. CI: 6.12–7.63). There were direct positive correlations between depression and negative health (rho: 0.284. *p* < 0.001), and between depression and taking antidepressants (rho: 0.271. *p* < 0.001). We also found increased OR and RR of suffering from depression (OR: 2.21 and RR: 2.07. *p* < 0.05) and taking antidepressants (OR: 1.86 and RR: 1.74. *p* < 0.05) in women compared to men (Table 6).

The older age group (elderly) also presented increased OR and RR of suffering from depression compared to the other age groups: young people (OR: 5.14. RR: 2.25), young adults (OR: 2.62. RR: 1.98) and older adults (OR: 1.32. RR: 1.24), all with a significance level of less than 0.05. Similarly, increased odds ratios and relative risks of taking antidepressants appeared in elderly vs. young (OR: 5.95. RR: 2.29), young adults (OR: 2.59. RR: 1.95) and older adults (OR: 1.28. RR: 1.21), with a significance level less than 0.05.

In relation to PA, the inactive persons presented increased risks of depression vs. walkers (OR: 1.39. RR: 1.28) and those low/medium PA levels (OR: 2.50. RR: 1.68) and high/very high PA levels (OR: 4.52. RR: 1.59), with a significance level less than 0.05. Direct correlations were also found between physical inactivity and the prevalence of depression (rho: 0.118. *p* < 0.001) in the inactive and high/very high PA level groups. There were similar findings for the prevalence of taking antidepressants, with increased risks in inactive persons vs. walkers (OR: 1.67. RR: 1.45) and those with low/medium PA levels (OR: 3.23. RR: 1.86) and high/very high PA levels (OR: 4.95. RR: 1.61), with *p* < 0.05 (Table 6).

## 4. Discussion

The study involved 17,141 people from the 2017 ENSE files, of whom 8942 were women and 8199 were men.

According to sex, significant differences (*p* < 0.05) were found between the proportions of men (5.9) and women (12.2), with a gap of 6.3 percentage points between the sexes. This is in line with the majority of studies indicating that the proportion of depression is higher in women than in men [25,26,27]. Another finding was the existence of dependency relationships between the prevalence of depression and sex (*p* < 0.001), as in the study by Pastor et al. where being female, among other factors, was found to be significantly associated with the risk of depression [28].

Similarly, significant differences (*p* < 0.05) were found between the proportions of antidepressant intake among men (3.3) and women (7.6), presenting a gap of 4.3 percentage points between the sexes, in line with the Campo-Barrientos study in which antidepressant consumption was found to be higher in women than in men [29], as well as the study of psychotropic drug consumption in Andalusia, where the prevalence of antidepressant consumption was found to be twice as high in women as in men [30]. Moreover, a dependence relationship was found between sex and antidepressant intake (*p* < 0.001), as in the study by Gil-García in which women were found to be three times more likely to consume antidepressants [30].

Seventy-eight percent of the population not diagnosed with depression presented positive self-perceived health, compared to 28.1% in those diagnosed with depression. This is in line with the results of another study, which concluded that those who perceive that they have a very good state of health have the lowest percentage of depression [31].

In contrast, the states of fair and negative reached higher prevalences in people with depression (42.8% and 29.1%) than in those not diagnosed (17.6% and 4.5%), with differences close to 25 percentage points, respectively. This is in line with the results of a study by Portellano-Ortiz, in which depressives showed poor perception of physical health [26].

Furthermore, significant differences were found between the prevalence of the different states of self-perceived health. In depressives, a higher prevalence of positive health was found in those with the highest levels of PA, and the lowest in inactive persons, with differences of up to 20 percentage points. The same trend was found in non-depressives. This is in line with the resuls of a study of patients with osteoarthritis [32], where self-perceived health was higher in active patients, or those of another study of young university students, where it was concluded that those with a higher level of PA had higher levels of self-perceived physical health [33].

The older age group (elderly) also presented increased OR and RR of suffering from depression, compared to the rest of the age groups. There is strong evidence in the scientific literature of a relationship between older age and higher levels of depression [34,35,36]. Similarly, increased odds ratios and relative risks of taking antidepressants appeared in older versus younger people (OR: 5.95): young (OR: 5.95. RR: 2.29), young adults (OR: 2.59. RR: 1.95) and older adults (OR: 1.28. RR: 1.21), which is in line with the results of the Henares-Montiel study [37].

The main finding concerned the relation to PA: inactive persons presented increased risks of depression vs. walkers (OR: 1.39. RR: 1.28), those with low/medium PA levels (OR: 2.50. RR: 1.68) and those with high/very high PA levels (OR: 4.52. RR: 1.59). Direct correlations were also found between physical inactivity and the prevalence of depression (rho: 0.118. *p* < 0.001). Similarly, in a study of patients with heart disease [38], a significant relationship was found between depression and physical inactivity. Similar results also occurred in the prevalence of taking antidepressants, with increased risks in inactive persons vs. walkers (OR: 1.67. RR: 1.45), those with low/medium PA levels (OR: 3.23. RR: 1.86) and those with high/very high PA levels (OR: 4.95. RR: 1.61), with *p* < 0.05. This is in line with the results of a previous study in which the probability of antidepressant intake was found to increase with inactivity [38].

### 4.1. Limitations

It should be noted that it there are a number of limitations to our study, such as those inherent to a survey: its cross-sectional nature and the fact that the data were obtained through information reported by the participants.

Cause–effect relationships cannot be established due to the methodology used in the article.

Only male and female sex were considered; non-binary sex was not taken into account.

Measures of physical activity were not evaluated objectively.

The diagnosis of depression was self-reported by the person participating in the study.

Only whether the participants were taking antidepressants was recorded; neither the active ingredient nor the amount taken by each participant who reported taking antidepressants was recorded.

The level of physical activity of people over 69 years of age was not recorded; no results were therefore obtained for people over 69 years of age.

### 4.2. Practical Implications

Possible initiatives that could be carried out through public health policies could be: the promotion of physical activity from primary care centers and through the inclusion of physical exercise professionals within the health service and in prevention services of public administrations, as well as creating programs that promote health-enhancing physical activity (HEPA) in mental health centers including multidisciplinary work by the exercise professional.

### 4.3. Future Lines of Research

Research with other types of designs that can establish cause and effect relationships, as well as provide information on optimal doses of PA to reduce the prevalence of depression and the use of antidepressants in the population may be useful. Moreover, comparing how the relationships found in this research have evolved during the pandemic and in future post-pandemic periods may also be a fruitful avenue for research.

## 5. Conclusions

It can be concluded that belonging to an inactive population group increases the risk of suffering from depression and of taking antidepressants. Walking could reduce the prevalence of depression in inactive people, although it would be advisable to incorporate programs of moderate and/or intense physical activity for a greater reduction in the prevalence of depression in the Spanish population.

## Figures and Tables

**Table 1:** Sociodemographic characteristics, prevalence of depression, use of antidepressants and level of physical activity in the Spanish population aged 18–69 years in 2017.

Variables				
Age (Years)	Total = 17141	Men = 8199	Women = 8942	*p*
Median (RI)	47 (21)	47 (20)	47 (21)	0.506
Mean (SD)	46.8 (13.3)	46.8 (13.2)	46.9 (13.3)	-
Age group (Years)	Total *n* (%)	Men *n* (%)	Women *n* (%)	*p* *
18–34	3297 (19.2)	1562 (19.1)	1735 (19.4)	0.242
35–49	6177 (36.0)	2993 (36.5)	3184 (35.6)	
50–64	5956 (34.7)	2860 (34.9)	3096 (34.6)	
65–69	1711 (10.0)	784 (9.6)	927(10.4)	
Depression	Total = 17137	Men = 8198	Women = 8939	*p* *
Yes *n* (%)	1572 (9.2)	480 (5.9) a	1092 (12.2) b	<0.001
No *n* (%)	15565 (90.8)	7718 (94.1) a	7847 (87.8) b	
Antidepressants	Total = 17138	Men = 8198	Women = 8940	*p* *
Yes *n* (%)	954 (5.6)	273 (3.3) a	681 (7.6) b	<0.001
No *n* (%)	16184 (94.4)	7925 (96.7) a	8259 (92.4) b	
Self-perceived health	Total = 17141	Men = 8199	Women = 8942	*p* *
Negative	1151 (6.7)	486 (5.9) a	665 (7.4) b	<0.001
Regular	3413 (19.9)	1439 (17.6) a	1974 (22.1) b	
Positive	12577 (73.4)	6274 (76.5) a	6303 (70.5) b	
Physical Activity Level	Total *n* (%)	Men *n* (%)	Women *n* (%)	*p* *
Inactive	2482 (14.5)	1156 (14.1)	1326 (14.8)	<0.001
Walkers	7901 (46.1)	3335 (40.7)	4566 (51.1)	
Low/Medium	4688 (27.3)	2350 (28.7)	2338 (26.1)	
High/Very High	2070 (12.1)	1358 (16.6)	712 (8.0)	

RI (Interquartile range); SD (Standard deviation); *n* (Number of participants); % (Percentage); Negative (They declared their health to be bad or very bad); Regular (They declared their health as regular); Positive (They declared their health as good or very good); IAF (Physical Activity Index); Inactive (IAF = 0; People who declare not to walk more than 10 min in a row). Walkers (IAF = 0; People who report walking more than 10 min in a row); Low/Medium (IAF between 1 and 30); High/Very High (FTI > 30); *p* (*p* value from Mann–Whitney U test); *p* * (*p*-value from chi-square test); ab (Each letters corresponds to significant differences between column proportions at 95% from pairwise z-test); Depression (reported or not to have been diagnosed with depression by a physician); Antidepressants (reported or not to have consumed antidepressants or stimulants in the two weeks prior to the survey).

**Table 2:** Relationship between the prevalence of depression and self-perceived health in the general Spanish population of the ENSE 2017 aged between 18 and 69 years, and according to the level of physical activity.

Self-Perceived Health: *n* (%)						
LPA	Depression	Total	Positive	Regular	Negative	*p*
Inactive (*n* = 2481)	Yes	355 (14.3)	62 (17.5) a	131 (36.9) a	162 (45.6) a	<0.001
	No	2126 (85.7)	1432 (67.4) b	465 (21.9) b	229 (10.8) b	
Walkers (*n* = 7899)	Yes	846 (10.7)	242 (28.6) a	379 (44.8) a	225 (26.6) a	<0.001
	No	7053 (89.3)	5247 (74.4) b	1464 (20.8) b	342 (4.8) b	
Low/Medium (*n* = 4687)	Yes	294 (6.3)	109 (37.1) a	125 (42.5) a	60 (20.4) a	<0.001
	No	4393 (93.7)	3681 (83.8) b	626 (14.2) b	86 (2.0) b	
High/Very High (*n* = 2070)	Yes	77 (3.7)	29 (37.7) a	38 (49.4) a	10 (13.0) a	<0.001
	No	1993 (96.3)	1774 (89.0) b	183 (9.2) b	36 (1.8) b	
Total (*n* = 17137)	Yes	1572 (9.2)	442 (28.1) a	673 (42.8) a	457 (29.1) a	<0.001
	No	15565 (90.8)	12134 (78.0) b	2738 (17.6) b	693 (4.5) b	

Depression (Whether or not they declared having been diagnosed with depression); *n* (Number of participants); % (Percentage); PAL (Physical activity level); IAF (Physical Activity Index); Inactive (IAF = 0; People who declared not going for a walk for more than 10 min at a time). Walkers (IAF = 0; People who report walking more than 10 min in a row); Low/Medium (IAF between 1 and 30); High/Very High (FWI > 30); *p* (*p*-value from chi-square test); ab (Different letters correspond to significant differences between column proportions in each NAF group and self-perceived health status at 95% from pairwise z-test).

**Table 3:** Relationship between the prevalence of anxiety and age group in the general Spanish population of the 2017 ENSE aged between 18 and 64 years, and by sex.

Age Group: *n* (%)							
Sex	Depression	Total	18–34 Age	35–49 Age	50–64 Age	65–69 Age	*p*
Men (*n* = 8198)	Yes	480 (5.9)	42 (2.7) a	145 (4.8) b	221 (7.7) c	72 (9.2) c	<0.001
	No	7718 (94.1)	1520 (97.3) a	2847 (95.2) b	2639 (92.3) c	712 (90.8) c	
Women (*n* = 8939)	Yes	1092 (12.2)	77 (4.4) a	277 (8.7) b	534 (17.3) c	204 (22.0) d	<0.001
	No	7847 (87.8)	1657 (95.6) a	2907 (91.3) b	2560 (82.7) c	723 (78.0) d	
Total (*n* = 17137)	Yes	1572 (9.2)	119 (3.6) a	422 (6.8) b	755 (12.7) c	276 (16.1) d	<0.001
	No	15565 (90.8)	3177 (96.4) a	5754 (93.2) b	5199 (33.4) c	1435 (83.9) d	
Sex	Antidepressants	Total	18–34 Age	35–49 Age	50–64 Age	65–69 Age	*p*
Men (*n* = 8198)	Yes	273 (3.3)	22 (1.4) a	87 (2.9) b	130 (4.5) c	34 (4.3) c	<0.001
	No	7925 (96.7)	1540 (98.6) a	2906 (97.1) b	2729 (95.5) c	750 (95.7) c	
Women (*n* = 8940)	Yes	681 (7.6)	38 (2.2) a	165 (5.2) b	342 (11.1) c	136 (14.7) d	<0.001
	No	8259 (92.4)	1697 (97.8) a	3019 (94.8) b	2752 (88.9) c	791 (85.3) d	
Total (*n* = 17138)	Yes	954 (5.6)	60 (1.8) a	252 (4.1) b	472 (7.9) c	170 (9.9) d	<0.001
	No	16184 (94.4)	3237 (98.2) a	5925 (95.9) b	5481 (92.1) c	1541 (90.1) d	

Anxiety (Whether or not they reported having been diagnosed with depression); Antidepressants (Whether or not they reported having taken antidepressants or stimulants in the two weeks prior to the survey); *n* (Number of participants); % (Percentage); *p* (*p*-value from chi-square test); abcd (Different letters correspond to significant differences between column proportions of the same row at 95% from pairwise z-test).

**Table 4:** Relationship between the prevalence of antidepressants and the level of physical activity in the general Spanish population of the ENSE 2017 aged between 18 and 69 years, and by sex.

Physical Activity Level: *n* (%)							
Sex	Depression	Total	Inactive	Walkers	Low/ Medium	High/Very High	*p*
Men (*n* = 8198)	Yes	480 (5.9)	118 (10.2) a	228 (6.8) b	97 (4.1) c	37 (2.7) d	<0.001
	No	7718 (94.1)	1038 (89.8) a	3107 (93.2) b	2252 (95.9) c	1321 (97.3) d	
Women (*n* = 8939)	Yes	1092 (12.2)	237 (17.9) a	618 (13.5) b	197 (8.4) c	40 (5.6) d	<0.001
	No	7847 (87.8)	1088 (82.1) a	3946 (86.5) b	2141 (91.6) c	672 (94.4) d	
Total (*n* = 17137)	Yes	1572 (9.2)	355 (14.3) a	846 (10.7) b	294 (6.3) c	77 (3.7) d	<0.001
	No	15565 (90.8)	2126 (85.7) a	7053 (89.3) b	4393 (93.7) c	1993 (96.3) d	
Sex	Antidepressants	Total	Inactive	Walkers	Low/Medium	High/Very High	*p*
Men (*n* = 8198)	Yes	273 (3.3)	77 (6.7) a	126 (3.8) b	47 (2.0) c	23 (1.7) c	<0.001
	No	7925 (96.7)	1079 (93.3) a	3208 (96.2) b	2303 (98.0) c	1335 (98.3) c	
Women (*n* = 8940)	Yes	681 (7.6)	174 (13.1) a	373 (8.2) b	111 (4.7) c	23 (3.2) c	<0.001
	No	8259 (92.4)	1152 (86.9) a	4192 (91.8) b	2226 (95.3) c	689 (96.8) c	
Total (*n* = 17138)	Yes	954 (5.6)	248 (10.1) a	499 (6.3) b	158 (3.4) c	46 (2.2) d	<0.001
	No	16184 (94.4)	2231 (89.9) a	7400 (93.7) b	4529 (96.6) c	2024 (97.8) d	

Depression (Whether or not they reported having been diagnosed with depression); Antidepressants (Whether or not they reported having taken antidepressants); *n* (Number of participants); % (Percentage); IAF (Physical Activity Index); Inactive (IAF = 0; People who reported not going for a walk for more than 10 min at a time). Walkers (IAF = 0; People who reported walking more than 10 min in a row); Low/Medium (IAF between 1 and 30); High/Very High (FWI > 30); *p* (*p*-value from chi-square test); abcd (Different letters correspond to significant differences between column proportions at 95% from pairwise z-test).

**Table 5:** Relationship between prevalence of depression, taking antidepressants and level of physical activity, according to age group in the general Spanish population of the 2017 ENSE aged between 18 and 69 years, and by age group.

Physical Activity Level: *n* (%)							
Age	Depression	Total	Inactive	Walkers	Low/Medium	High/Very High	*p*
18–34 years (*n* = 3296)	Yes	119 (3.6)	23 (5.5) a	56 (4.6) a	33 (3.3) a	7 (1.1) b	<0.001
	No	3177 (96.4)	398 (94.5) a	1162 (95.4) a	975 (96.7) a	642 (98.9) b	
35–49 years (*n* = 6176)	Yes	422 (6.8)	103 (11.0) a	202 (7.9) b	90 (4.9) c	27 (3.3) c	<0.001
	No	5754 (93.2)	832 (89.0) a	2363 (92.1) b	1760 (95.1) c	799 (96.7) c	
50–64 years (*n* = 5954)	Yes	755 (12.7)	173 (19.7) a	418 (13.4) b	126 (8.8) c	38 (7.4) c	<0.001
	No	5199 (87.3)	704 (80.3) a	2705 (86.6) b	1313 (91.2) c	477 (92.6) c	
65–69 years (*n* = 1711)	Yes	276 (16.1)	56 (22.6) a	170 (17.1) b	45 (11.5) c	5 (6.3) c	<0.001
	No	1435 (83.9)	192 (77.4) a	823 (82.9) b	345 (88.5) c	75 (93.8) c	
Total (*n* = 17137)	Yes	1572 (9.2)	348 (13.7) a	788 (9.8) b	320 (6.5) c	83 (3.7) d	<0.001
	No	15565 (90.8)	2185 (86.3) a	7279 (90.2) b	4567 (93.5) c	2144 (96.3) d	
Age	Antidepressant	Total	Inactive	Walkers	Low/Medium	High/Very High	*p*
18–34 years (*n* = 3237)	Yes	60 (1.8)	20 (4.8) a	26 (2.1) b	8 (0.8) c	6 (0.9) b,c	<0.001
	No	3237 (98.2)	401 (95.2) a	1193 (97.9) b	1000 (99.2) c	643 (99.1) b,c	
35–49 years (*n* = 6177)	Yes	252 (4.1)	73 (7.8) a	116 (4.5) b	53 (2.0) c	10 (1.2) d	<0.001
	No	5925 (95.9)	862 (92.2) a	2449 (95.5) b	1798 (97.1) c	816 (98.8) d	
50–64 years (*n* = 5953)	Yes	472 (7.9)	118 (13.4) a	259 (8.3) b	69 (4.8) c	26 (5.0) c	<0.001
	No	5481 (92.1)	760 (86.6) a	2863 (91.7) b	1369 (95.2) c	489 (95.0) c	
65–69 years (*n* = 1711)	Yes	170 (9.9)	40 (16.1) a	98 (9.9) b	28 (7.2) b	4 (5.0) b	<0.001
	No	1541 (90.1)	208 (83.9) a	895 (90.1) b	362 (92.8) b	76 (95.0) b	
Total (*n* = 17716)	Yes	954 (5.6)	251 (10.1) a	499 (6.3) b	158 (3.4) c	46 (2.2) d	<0.001
	No	16184 (94.4)	2231 (89.9) a	7400 (93.7) b	4529 (96.6) c	2024 (97.8) d	

Depression (Whether or not they declared having been diagnosed with depression); Antidepressants (Whether or not they declared having taken antidepressants or stimulants); *n* (Number of participants); % (Percentage); IAF (Physical Activity Index); Inactive (IAF = 0; People who declared not going for a walk for more than 10 min at a time). Walkers (IAF = 0; People who reported walking more than 10 min in a row); Low/Medium (IAF between 1 and 30); High/Very High (FWI > 30); *p* (*p* value from chi-square test); abcd (Different letters correspond to significant differences between column proportions at 95% from pairwise z-test).

**Table 6:** In the Spanish population aged 18–69 years: Ratio of probability and relative risk of having a negative perception of health, whether or not suffering from depression, or taking antidepressants; probability and relative risks of suffering from depression and taking antidepressants, according to age group; and level of physical activity.

Risks of having negative self-perceived health								
Depression Condition		OR	CI95%	RR	CI95%	*p* *	rho	*p*
Depression	No Depression	8.80	7.70–10.05	6.53	5.87–7.26	<0.001	0.284	<0.001
Antidepressants	No Antidepressants	9.92	8.53–11.54	6.84	6.12–7.63	<0.001	0.271	<0.001
Risk of depression								
Sex		OR	CI95%	RR	CI95%	*p* *	rho	*p*
Women	Men	2.21	1.97–2.47	2.07	1.86–2.29	<0.001	0.106	<0.001
Age Group		OR	CI95%	RR	CI95%	*p* *	rho	*p*
65–69 years	18–34 years	5.14	4.11–6.42	2.25	2.08–2.43	<0.001	0.22	<0.001
	35–49 years	2.62	2.23–3.09	1.98	1.79–2.20	<0.001	0.135	<0.001
	50–64 years	1.32	1.14–1.54	1.24	1.11–1.38	<0.001	0.042	<0.001
50–64 years	18–34 years	3.88	3.18–4.73	1.39	1.35–1.44	<0.001	0.120	<0.001
	35–49 years	1.98	1.75–2.24	1.35	1.29–1.42	<0.001	0.099	<0.001
35–49 years	18–34 years	1.96	1.59–2.41	1.21	1.16–1.27	<0.001	0.066	<0.001
Physical Activity Level IAF Level		OR	CI95%	RR	CI95%	*p* *	rho	*p*
Inactive	Walkers	1.39	1.22–1.59	1.28	1.16–1.40	<0.001	0.048	<0.001
	Low/Medium	2.50	2.12–2.94	1.68	1.55–1.81	<0.001	0.133	<0.001
	High/Very High	4.32	3.35–5.57	1.59	1.51–1.68	<0.001	0.180	<0.001
Walkers	Low/Medium	1.79	1.56–2.06	1.20	1.16–1.25	<0.001	0.075	<0.001
	High/Very High	3.11	2.45–3.94	1.18	1.15–1.20	<0.001	0.098	<0.001
Low/Medium	High/Very High	1.73	1.34–2.24	1.15	1.10–1.22	<0.001	0.052	<0.001
Risks of antidepressant use								
Sex		OR	CI95%	RR	CI95%	*p* *	rho	*p*
Women	Men	1.86	1.68–2.05	1.74	1.59–1.90	<0.001	0.093	<0.001
Age Groups		OR	CI95%	RR	CI95%	*p* *	rho	*p*
65–69 years	18–34 years	5.95	4.41–8.04	2.29	2.10–2.50	<0.001	0.184	<0.001
	35–49 years	2.59	2.12–3.18	1.95	1.72–2.21	<0.001	0.107	<0.001
	50–64 years	1.28	1.07–1.54	1.21	1.05–1.38	<0.01	0.030	<0.01
50–64 years	18–34 years	4.65	3.54–6.10	1.41	1.36–1.46	<0.001	0.126	<0.001
	35–49 years	2.03	1.73–2.37	1.36	1.28–1.44	<0.001	0.081	<0.001
35–49 years	18–34 years	2.30	1.73–3.05	1.25	1.18–1.32	<0.001	0.060	<0.001
Physical Activity Level IAF Level		OR	CI95%	RR	CI95%	*p* *	rho	*p*
Inactive	Walkers	1.67	1.42–1.96	1.45	1.30–1.61	<0.001	0.063	<0.001
	Low/Medium	3.23	2.63–3.96	1.86	1.71–2.02	<0.001	0.138	<0.001
	High/Very High	4.95	3.59–6.82	1.61	1.52–1.71	<0.001	0.159	<0.001
Walkers	Low/Medium	1.93	1.61–2.32	1.22	1.17–1.28	<0.001	0.064	<0.001
	High/Very High	2.97	2.19–4.03	1.17	1.13–1.20	<0.001	0.073	<0.001
Low/Medium	High/Very High	1.54	1.10–2.14	1.12	1.04–1.21	<0.05	0.031	<0.05

OR (Odds ratio); CI (Confidence Interval); RR (Relative risk); PAI (Physical Activity Index); Inactive (PAI = 0; Reported not walking more than 10 min in a row); Insufficient (IAF = 0; They reported walking more than 10 min in a row); Low (IAF between 1 and 15); Medium (IAF between 16 and 30). High (FWI between 31 and 45); Very High (FWI greater than 45); *p* (*p*-value); rho (Spearman correlation coefficient); *p* * (*p*-value from chi-square test); Depression (reported having been diagnosed with depression); No depression (reported not having been diagnosed); Antidepressants (Reported taking antidepressants or stimulants); Antidepressants (Reported taking antidepressants or stimulants in the two weeks prior to the survey); No antidepressants (Reported not taking antidepressants or stimulants in the two weeks prior to the survey).

## Data Availability

The data used were obtained from public use files, which are available on the website of the Spanish Ministry of Health, Consumer Affairs, and Social Welfare: https://www.sanidad.gob.es/estadEstudios/estadisticas/EncuestaEuropea/Enc_Eur_Salud_en_Esp_2020.htm (accessed on 5 November 2021).
